# The comparative study by Raman spectroscopy of the plastic tide in the three ports of the Mediterranean Sea

**DOI:** 10.1007/s11356-023-30973-z

**Published:** 2023-11-24

**Authors:** Agnieszka Dąbrowska, Seweryn Kipa, Michalis Vasilopoulos, Magdalena Osial

**Affiliations:** 1 University of Warsaw, Faculty of Chemistry, Laboratory of Spectroscopy of Intermolecular Interactions, Pasteura 1, 02-093 Warsaw, Poland; 2 University of Warsaw Biological and Chemical Research Centre, Żwirki i Wigury 101 st, 02-089 Warsaw, Poland; 3 Institute of Fundamental Technological Research, Polish Academy of Sciences, Pawińskiego 5B, 02-106 Warsaw, Poland

**Keywords:** Raman spectroscopy, Marine microplastics, Mediterranean Sea, Polymer leakage, Electrochemical analysis

## Abstract

This paper summarizes the field studies on marine microplastics (MPs) carried out in the autumn season in four various localisations within three ports chosen at the Mediterranean Sea near the French Riviera and the West Coast of Italy (within the Ligurian Sea). It considers the transport problem and the fate of the MPs introduced to the sea by analysing beach debris found on the shore after the stormy weather. Monitored ports included Saint-Tropez, Portoferraio and Porto Ercole, in which two different places were monitored. The aim is to approach the plastic tide phenomena by concentrating on a selected fraction of all MPs presented on the seashore. The final identification of debris was performed using Raman spectroscopy, providing a high-resolution signal. The PE, PP and PS contents were compared as the most frequent and representative polymers. Finally, we tackle the pending issue of the compound leakage from the MPs taking the environmentally aged particles from Portoferraio for further laboratory experiments and discuss an innovative approach with a low detection limit based on the electrochemical methods.

**Graphical Abstract:**

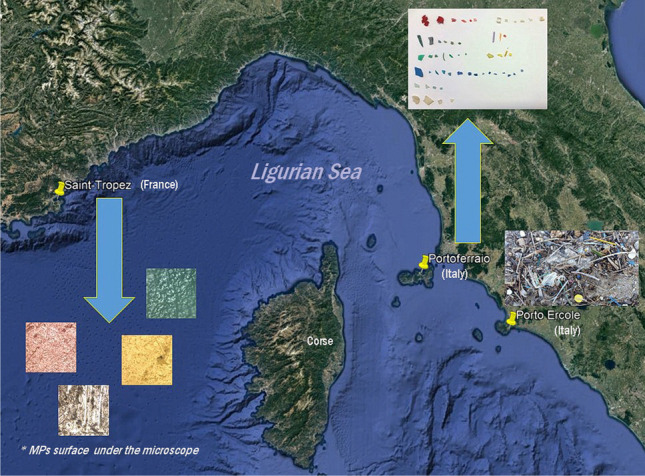

**Supplementary Information:**

The online version contains supplementary material available at 10.1007/s11356-023-30973-z.

## Introduction

### Microplastics in the Mediterranean Sea — short overview of the state of the art and current directions in studies

The coasts of the Mediterranean Sea are densely populated areas with 10% of the global coastal inhabitants. Therefore, the marine environment is constantly flooded with marine litter, of which the most significant proportion is related to the presence of synthetic materials (Martellini et al. 2018). All their fragments smaller than 5 mm are called microplastics (MPs). There has been an increase in studies conducted on the MPs’ pollution in the last decades, but the data is still insufficient to create a comprehensive model of MPs’ transportation and precisely determine their amount (Simon-Sánchez et al. 2022). For example, river mouths collect large concentrations of litter, and by proxy plastics, they run through large areas for hundreds of kilometres, accumulating plastics. Thus, the studies in deltas of main rivers are particularly important for detecting accumulation hot spots (Simon-Sánchez et al. 2019; Constant et al. 2020; Pellegrini et al. 2023). However, there is no data on MP pollution from river mouths such as the Nile River or Barden, Geoksu and Seyhan located in the Mersin Bay area at the eastern end of the Mediterranean (Martellini et al. 2018). The studies on the Mediterranean Sea frequently analyse samples from sediments (Li et al. 2023), waters (Nunes et al. 2023), beaches (Şener and Yabanlı 2023), marine organisms and seafood (Kibria 2023).

Seafloor litter is the least exploited component of marine litter. The principal means for collecting seafloor litter data is trawling for fishing, which needs to be replaced with methods with a low environmental footprint. The study in Cyclades, Greece, using underwater imagery (Fakiris et al. 2022)⁠, assessed the spatiotemporal distribution and composition of seafloor litter in shallow coastal environments. Plastic items smaller than 1 mm were collected in the sediments of nine beaches spanning 124 km on the Algerian coast (Taïbi et al. 2021). From all 777 plastic samples collected, 83.27% were micro and macro plastic fragments, 14.93% were microplastic pellets, and 1.03% was filmed. Higher plastic pollution was noticed near coastal villages, indicating increased pollution due to human coastal activity such as seaside tourism and recreational activities. Numerous floating plastic items in the Bay of Marseille had ecotoxicological implications (Gérigny et al. 2022).

Researchers in Spain (Asensio-Montesinos et al. 2021) examined litter from five cobble beaches over 3 months in winter. They found out that 77% of the materials gathered were plastic. The leading litter dynamic drivers were storms that accumulated floating plastic items such as bottles on remote beaches. Other research (Compa et al. 2022), executed within the Cabrera Archipelago Maritime-Terrestrial National Park in the Balearic Islands, reported the beach’s spatial distribution and physical characteristics of macro-and micro-litter. The macro-litter data showed that 1.9 items·m^−2^ were found, with twice as many items found during the low tourism season compared to the high tourism season. The micro-litter data showed an average of 13,418 items·m^−2^. The most common polymer types were PE (64%) and PP (17.2%). The most abundant type of material for macro and microplastic litter was plastic (90%). Seasonal changes are often correlated with heavy storms and additional flux of materials directly from the high seas instead of the expected land-sea direction of flux. Thus, reversed transportation is often called a plastic tide, when MPs are brought back on shore.

Moreover, the Mediterranean seagrass soils (Dahl et al. 2021) efficiently trap organic and inorganic particles. For the seagrass from three locations along the Spanish Mediterranean coast, precisely in the Almeria region, known for its agricultural industry, and at Cabrera Island (Santa Maria), microplastic accumulation was almost non-existent until the mid-1970s. Their study showed that intense anthropogenic activity in the Almeria region is directly linked to high plastic contamination. A recent study (Fagiano et al. 2022) showcases a study that described microplastic pollution in Cabrera Marine Protected Aream’s coastal waters and an insight into the study of zooplankton community composition within coastal MPAs depending on the distance from the coastline. There was no inshore-offshore gradient detected in the study. MPAs are not protected from plastic contamination, and no clear trend between sediment grain size and microplastic deposition in sediments was found (Alomar et al. 2016). However, the sediments collected from MPAs contained the highest concentration of MPs, once again indicating that MPA beaches act as sinks of marine litter (Fagiano et al. 2022).

So far, the open questions related to the microplastic pollution in the Mediterranean are related to their flux (Valente et al. 2023) and transport modelling (Ourmieres et al. 2023), seasonal monitoring (Provenza et al. 2022), quantification (Caldwell et al. 2022), detection, separation (Zhao et al. 2022) and classification that is still a challenge (Priya et al. 2022). Ecotoxicological issues are not neglected, as MPs impact on sea urchins (Raguso et al. 2022), (Digka et al. 2023), striped dolphins (Novillo et al. 2020) and swordfish (Di Giacinto et al. 2023), and mollusc-human transfers (Expósito et al. 2022) are extensively studied problems.

Overall, it seems crucial to characterize qualitatively and quantitatively the polymer debris in the environment. Moreover, so far, no attempts have been reported for the Mediterranean Sea to tackle the issue of a plastic tide. Namely, to distinguish the MPs’ origin: those from the shore and those taken back on land from high seas by currents or storm tides. Thus, this paper aims to concentrate mainly on larger MP fractions (1–5 mm) that were identified as being carried by the autumn and winter storms. The collected material is identified based on the high-resolution Raman spectroscopy being an efficient method and the standard in plastic characterization (Xu et al. 2019) with a higher spatial resolution than a complementary and more popular being cheaper, FTIR (*Fourier transform infrared spectroscopy*).

Among many pending issues related to the microplastics in the Mediterranean Sea, we decided to focus on the plastic tide phenomena. One may point out that not all plastic pollution observed in a particular area is related to local sources. In many remote and pristine zones of the Earth, the microplastics were carried by currents and water flux. Those “imported” ones usually exhibited different sorptive and physical properties than those already classified and studied locally. Thus, it started to be particularly important to distinguish between the debris from local land sources and marine microplastics from high seas. Since 2019, sampling for this research has occurred, and this problem has been intensively studied (Asensio-Montesinos et al. 2022) by many groups worldwide. Herein, we contribute to this picture by the plastic pollution evidence from four harbours. We aimed to regard the MPs’ often neglected source. Thus, the debris is thrown back onshore as a plastic tide. Concentrating mainly on that fraction, we want to approach the problem of MPs’ circulation patches and complex transport in the ocean system. The ports selected for monitoring included Saint-Tropez, a well-known tourist destination; the unique Portoferraio, but still an essential seasonal destination; and Porto Ercole. In addition, with this choice of harbours, we monitored the triangle around and within the Santuario Pelagos — one of the most important marine protected areas with the highest concentration of cetaceans.

#### Leakage from microplastics and its detection

Since microplastic lands in an environment are exposed to natural conditions like UV light, oxygen, salts, water and many other factors, it releases many chemicals, including dyes. Therefore, it is necessary to monitor their level in the aquatic systems. However, despite its wide application for removing and monitoring different chemical compounds also adsorbed on MPs (Guerrini et al. 2021), there is still a lack of broad application in detecting the compounds released from microplastics. The problem is complex, as in the case of landfilled leachat (Teng et al. 2021). Therefore, electrochemical tools were proposed in this work for detecting the chemicals released from microplastics complementary to other techniques. This approach’s main advantage is finding the solution to the low detection limit problem. Electrochemical techniques such as voltammetry or amperometry can be used for fast and sensitive detection of several molecules present in aqueous solutions, such as dyes. In this work, we used cyclic voltammetry to detect chemical compounds leaching from microplastics within weathering in laboratory conditions as a complementary technique to the Raman spectroscopy used to determine solid samples’ chemical composition.

## Materials and methods

### Sampling sites and protocol

After three consecutive storm days, samples were collected in the autumn and winter seasons (mid-October 2019–mid-February 2020). More than 70 h of storm guarantee efficient material mixing within the tidal zone. The idea was to focus on the material thrown back on shore from the sea. Thus, one can detect MPs being a part of a so-called plastic tide, namely particles not dropped initially on the seacoast where they are finally found being transported from high seas. All sites were chosen randomly on the beach within their flooded tide zone (batching seawater), where there were previously no visible plastic fragments. That was to ensure they appeared after the storm, probably taken from different regions.

Each debris was collected with tweezers from a standard 1-m square. The subset of samples that exhibited at least 50% of selected MP classes in at least 50% of other sets was chosen from several to represent this localization. The three ports, Saint-Tropez, Portoferraio and Porto Ercole, were chosen to represent Santuario Pelagos marine protected area. In particular, the part of the French Riviera, Elba (island), is under anthropogenic pressure due to enhanced tourism and is susceptible to wave passage part of the West Italian coastline. Two different sampling localisations were chosen in Porto Ercole (Figure S1). They correspond to two entirely different water dynamics within the harbour (constant flux and isolated lagoon).

The detail coordinates are the following: 42° 48′ 6.29″ N, 10° 19′ 33.23″ E for Portoferraio, 43° 16′ 24.85″ N, 6° 38′ 19.00″ E for Saint Tropez, 42° 23′ 40.37″ N, 11° 12′ 18.93″ E for Porto Ercole (1) and 42° 23′ 26.03″ N, 11° 12′ 29.31″ E for Porto Ercole (2).

MPs were collected in the 1–5-mm size range, only if a similar bigger fragment was seen in their vicinity. That was to ensure that they are from the secondary source type and fragmented already in the marine environment, not before or being the primary MPs. Moreover, this methodology was chosen to provide insight into the plastic tide phenomena, as particles transported from the high seas were reported to be usually sets of debris with different sizes from fragmented original particles. Separating the original on-shore plastic from the particles brought by sea and storm waters is impossible with a lower size fraction. Moreover, the initial monitoring of MPs’ presence and lack in a particular area was done visually, so only the visible fraction can be assumed not to be presented initially on the beach.

The general knowledge of sampling protocols has developed substantially during the last 3 years, limiting this approach. Thus, we propose some corrections and recommendations for future research presented in a discussion. Google Earth Pro software was used for KLM files and organizing all monitoring data.

### Raman spectroscopy of microplastics

All collected spectra were registered on DXR Raman Microscope (Thermo Scientific) with the four available lase lines: 455, 532, 633 and 780 nm. When possible, the standard green line was used for the signal acquisition. However, in cases of dominant auto-fluorescence, all other lines were adopted starting from 780 nm. Several points were tested within each debris in order to provide the best signal-to-noise ratio. Spectra were collected mainly with the 50-μm aperture, the lens of 10-mm-focal length, with laser power ~10 mW. The standard OMNIC background cutoff was used. Samples were not chemically purified by mechanical separation and rinsing on a filter to maintain their morphology and surface structure unaffected.

### Electrochemistry

Electrochemical measurements were performed in the conventional three-electrode system using the Autolab potentiostat-galvanostat (Eco-ChemieNetherlanden) with GPES software. The glassy carbon (GC) was used as a working electrode, while its surface was modified with the polyindole (PIN) nanobrush decorated with Au nanoparticles (NPs). Then, the PIN-Au NP electrode improved sensing compared to the bare GC. The PIN-Au NP layer onto the GC electrode was prepared with the procedure described in Warczak et al. (2021). The surface area for GC was 0.0314 cm^2^. A saturated Ag|AgCl|KCl electrode was applied as the reference electrode, and a Pt mesh was used as a counter electrode. The microplastic samples were washed with ethanol to remove organic impurities and then with Mill-Q quality water. Solid samples were ground mechanically, weathered in Milli-Q water for 10 months, and stored in glass jars with an exposition on the daylight at room temperature. Then, the 0.1 M LiClO4 (≥ 98%, Sigma-Aldrich) was added as an essential electrolyte to the particular solution gained from the weathered microplastic samples. All electrochemical experiments were performed in the argon atmosphere, where the solution was purged for 30 min to remove oxygen, and then the nitrogen was purged continuously above the solution. Additionally, the organic dye solutions of over 15 different compounds with the 1 ppm concentration were diluted in Milli-Q water to record the electrochemical signals as a reference towards the characterization of the weathered solutions. The morphology studies were performed with a Scanning Electron Microscopy FE-SEM Merlin (Zeiss, Germany) working at a low beam voltage (3 kV) and a sample current (15–30 pA) to reveal the detailed images of the tested samples.

### Samples’ initial preparation for leakage studies and reference materials

The samples from Portoferraio (Table S1) were chosen for laboratory ageing and leakage monitoring. That was due to their visible natural deterioration. First, we crushed our probes to increase the surface-to-volume ratio further to obtain the most accurate measurements as if they were performed in natural waters containing microplastic particles. After plastic preparation, we put fragments of each inside glass vials, added milli-Q water (resistance 18.2 MΩ^.^cm), and stirred such mixtures on a magnetic stirrer to simulate natural conditions in which such particles could be placed. All probes were left for 1 week, being constantly stirred. Such long-term stirring was performed considering rinsing pollutants. The obtained contaminated water was further analysed by Raman spectroscopy and electrochemically.

## Results and discussion

### Spectral Identification of MPs

**Fig. 1:**
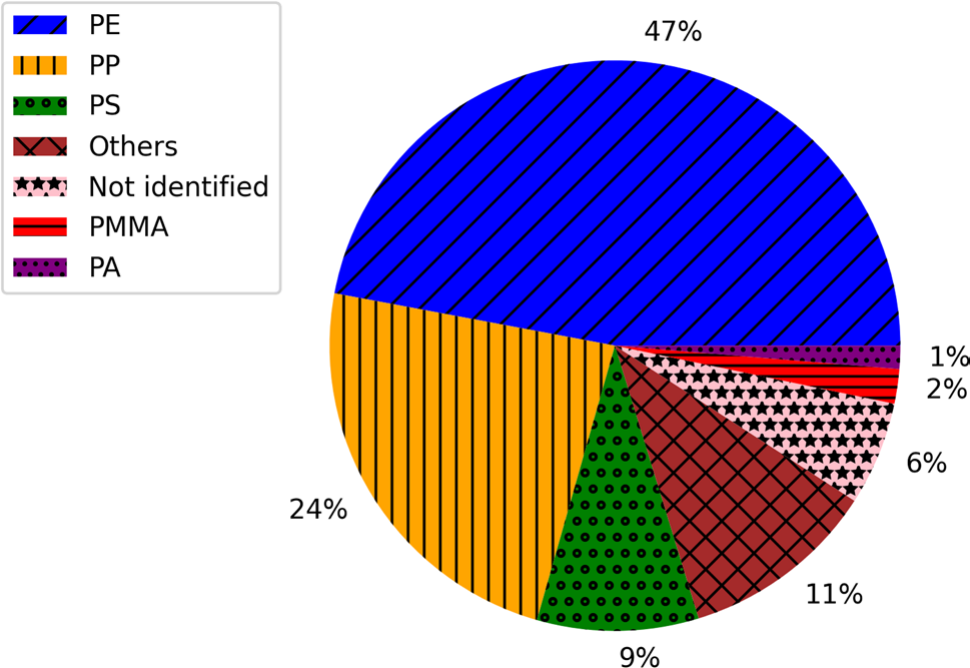
The qualitative identification (by Raman spectroscopy) of all samples in three ports and four localizations

The representative debris collected in all selected localizations was efficiently identified in 94% by Raman spectroscopy (Table S2) with dominant content of the polyethylene (PE), polypropylene (PP) and polystyrene (PS) equal to 47%, 24% and 9%, respectively (Fig. 1). Using one approach is favourable and was possible due to the manual treatment of all fragments (each one was analysed separately by the operator), high resolution of spectra, substantial signal to noise ratio, and in a few cases the measurements with more than just one (532 nm) laser line. As high-resolution spectra were registered, they provided much more information than is obtained in popular semi-automatic protocols or using the fast mapping of a whole filter.

**Fig. 2:**
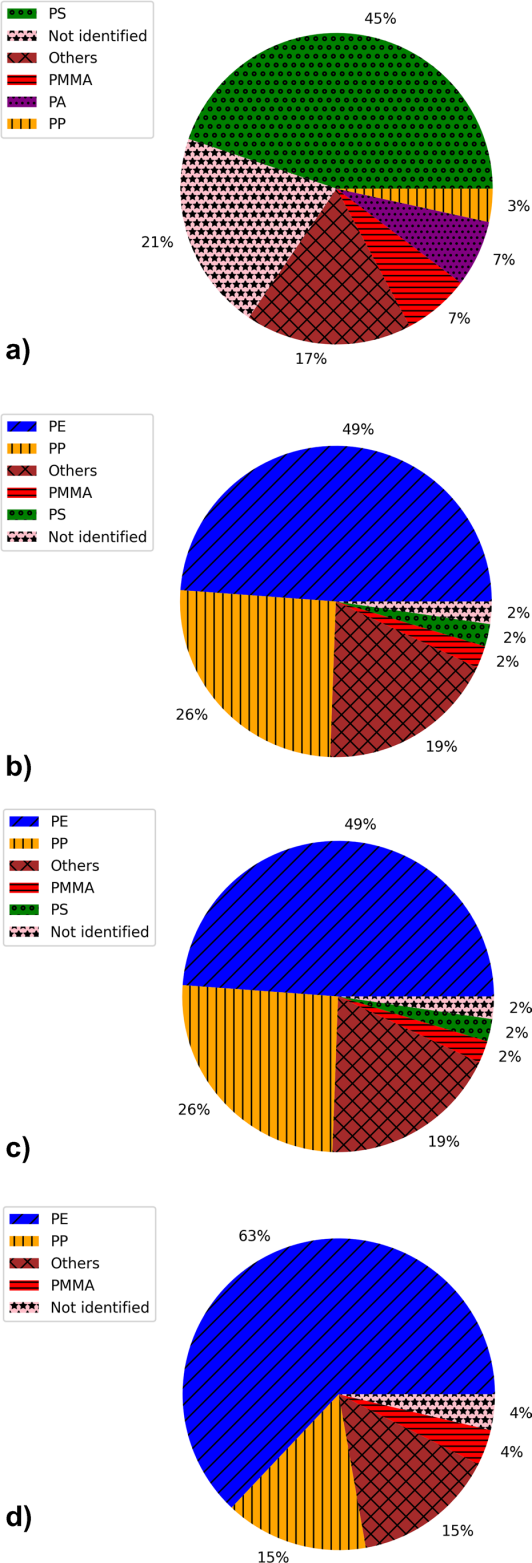
The relative content of particular polymer types in selected localizations: **a** Porto Ercole (1), **b** Porto Ercole (2), **c** Portoferraio and **d** Saint-Tropez

**Fig. 3:**
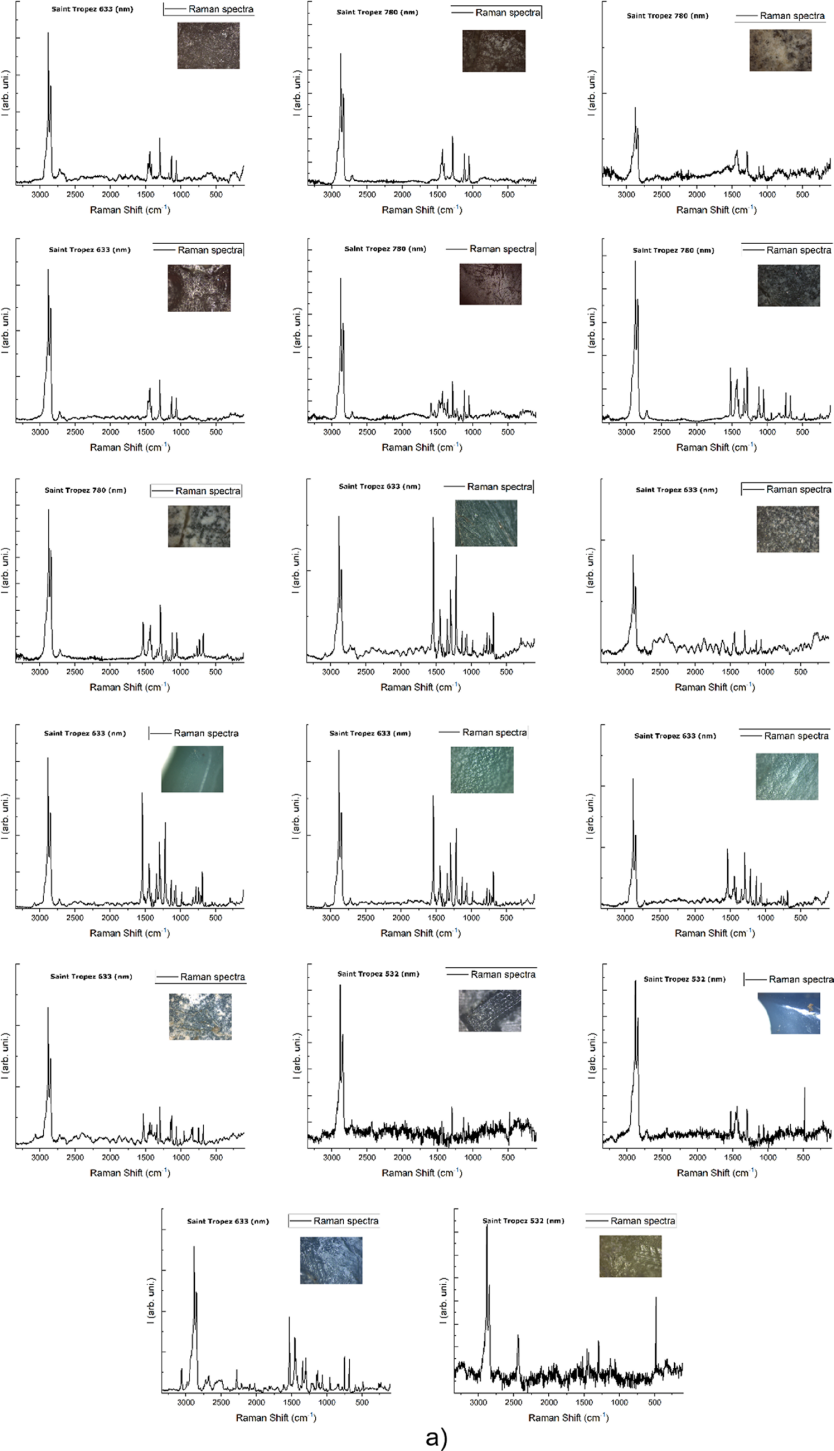

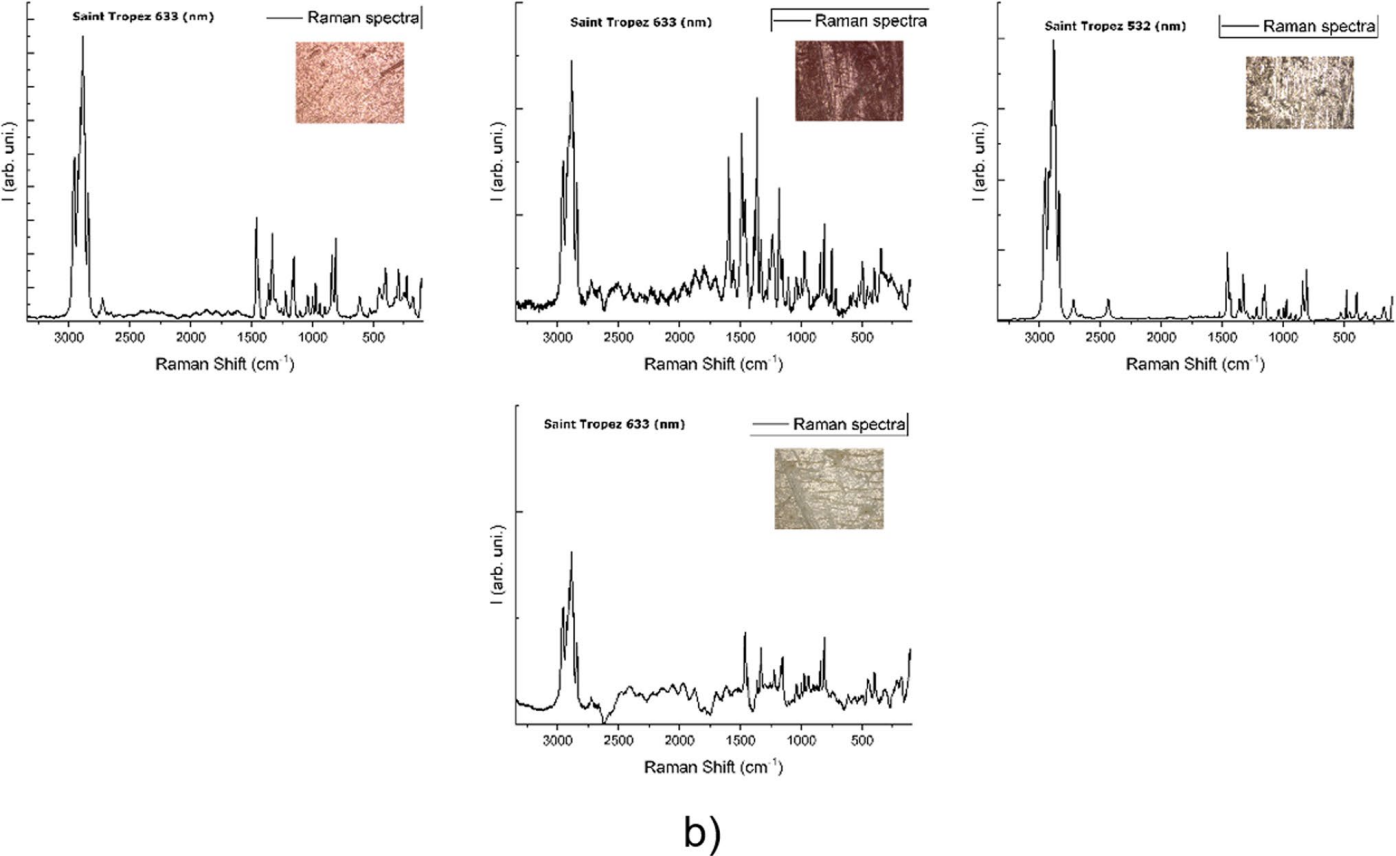
An overview of Raman spectra for the most numerous samples collected in Saint-Tropez: **a** PE and **b** PP

Although the PP, PE and PS were the most numerous in all localizations, one can observe some differences between ports in their overall polymer type composition (Fig. 2). For instance, the PS was more abundant in Porto Ercole (1). Porto Ercole (1) and (2) exhibited higher differences in polymer types than Saint-Tropez and Portoferraio. Figures 3, S2, S3 and S4 collect Raman spectra and microscopic pictures of analysed debris. One may note the differences in ageing level, morphology, roughness and shape even in the case of the same type of material.

The results are consistent with previous research in this area. For instance, a monitoring survey identifying microplastic particles in the Lagoon of Venice in Italy sediments from ten selected sampling sites revealed that MP pollution is present throughout the Lagoon. In addition, there were 14,452 synthetic materials identified as synthetic polymers, and 82% of these were polyethylene and polypropylene polymers. The study concluded that MP tends to accumulate in low-dynamic areas and can potentially transfer to the locally harvested Manila clams, *Ruditapes philippinarum*, distributed worldwide, with an annual production level reaching 40,000 t (Vianello et al. 2013)⁠. On the Southern Mediterranean coasts, sediments and seaworms, *Hediste diversicolor*, were examined to find MPs (Missawi et al. 2020), to identify the accumulation of MP and their potential to be transferred through the aquatic food chain. Most polymers were polyethylene (PE) and polypropylene (PP), with a size between 3 and 1.2 μm. The studies showed high microplastic abundance in those sites. The number of synthetic pollutants found in seaworm samples strictly correlated with the number of pollutants found in the sediments of the sites. The affected seaworms showed increased changes in biomarkers of cytotoxicity and neurotoxicity, according to microplastic tissue concentration, indicating their potentially harmful effect on the organism.

In the Northern Tyrrhenian, a survey was executed (Mistri et al. 2020) to investigate MP contamination of seafloor along a 31-km-long transect from the port of Piombino in Tuscany to the port of Portoferraio on Elba Island (from which two sample sites were analysed in our study). The Tyrrhenian Sea strip was chosen as a busy shipping route connecting the mainland to Elba Island. A total of 58 sediment samples were analysed, and almost all contained plastics. Microplastics accounted for 84.5% of the total amount found; mesoplastics comprised 13.2%; and macroplastics comprised only 2.3%. The collected particles were categorized as filaments, film and fragments, consisting of 74%, 17% and 9%, respectively. No primary microplastics like granules or pellets were found. They identified the MP with FTIR spectroscopy, which evidenced the presence of six types of polymers, such as nylon, polyethylene and polyurethane, which are all frequently used in a wide range of domestic and marine applications, indicating a broad spectrum of sources.

Another research team (Baini et al. 2018) studied the abundance of microplastics in the coastal waters of Tuscany. They analysed 24 samples from 16 sampling stations placed 0.5, 5, 10 and 20 km from the shoreline in four different locations. The area investigated is characterized by many different anthropogenic pressures, such as the Leghorn port, one of the largest Italian ports, one of the largest Italian rivers, the Amo river, and other minor rivers that cross several agricultural sites and industrial areas. Samples were collected from the water surface and column using manta trawls as deep as 100 m. Out of the 24 samples, a total of 1586 microplastics were isolated. The analysis shows that the sampling area and season do not affect MPs’ abundance. However, a trend showed an increased concentration of samples found in the more distant sample sites (20 and 10 km). It is speculated that this trend is observed due to the hydrodynamic of the area, such as near-shore circulation, which might transport the marine litter far from the shore. The most frequent size was 1–2.5 mm, and the most abundant polymers were polyethylene (66%), followed by polypropylene (28%) and polystyrene (5%). PE, PP and PS dominated also among the floating microplastics (Kedzierski et al. 2022).

**Fig. 4:**
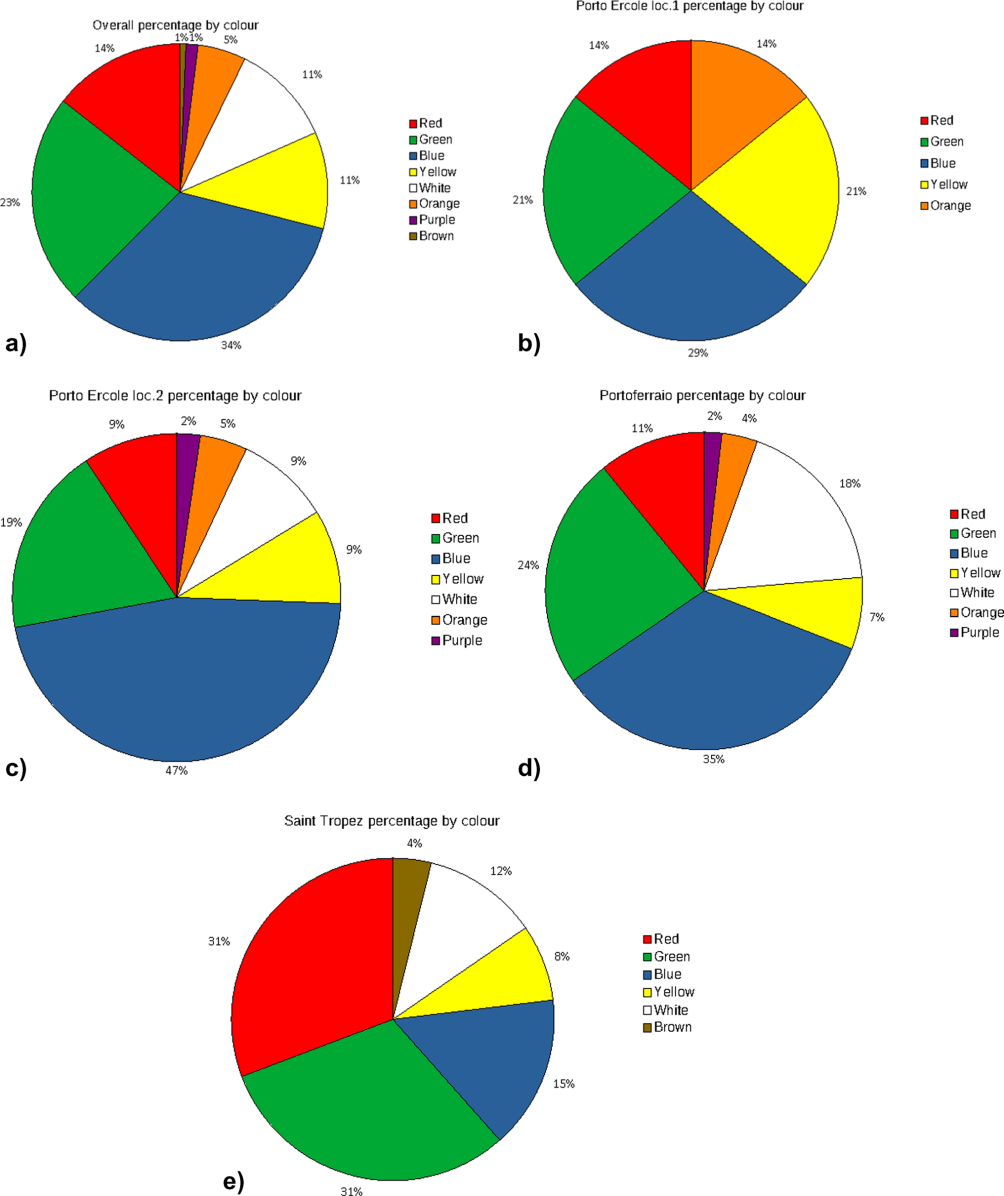
The summary of colour percentage in **a** overall and in **b** Porto Ercole (1), **c** Porto Ercole (2), **d** Portoferraio and **e** Saint-Tropez

Also, colour differences vary between ports (Fig. 4). However, blue and green fractions are usually in the majority and constitute > 50% of MPs. That is coherent with previous research in those areas and particularly alarming as dark colours enhance animals’ swallows.

MPs’ deterioration is one indication of their plastic tide origin. Usually, the MPs are less degraded near their source (place of entering the environment). Moreover, the leakage is more probable due to the gradual natural weathering of materials. As an extension of this approach, the enhanced sampling protocol is recommended, with seasonal changes included. That was the main limitation of this study and precluded the more general conclusions. However, one may consider it an advantage for fast monitoring to provide a general overview of the situation.

Finally, the different ageing levels of PE can be observed on the Raman spectra comparing the CH_2_:CH_3_ intensity ratio. For instance, comparing the relative intensities between PE bands (Fig. 3a) at 2882 and 2848 cm^−1^, for asymmetric and symmetric –CH_2_ stretching, one may conclude about considerably different ageing levels of MPs even at the exact localization. NO previously reported research considered the difference between plastic tide MPs and those from land-based sources. It would be interesting to compare the weathering levels between those two groups.

### The preliminary approach to the monitoring of leakage

In samples 3 and 13, the new bands in milli-Q water were also observed on the Raman spectra (Fig. S5), confirming the leakage from PP and PE, respectively. Unfortunately, the band 930 cm^−1^ cannot be unequivocally subscribed to a particular added compound. In samples aged in the milli-Q water for over 10 months, the small fragments were abundant and present in the liquid (Fig. S6).

**Fig. 5:**
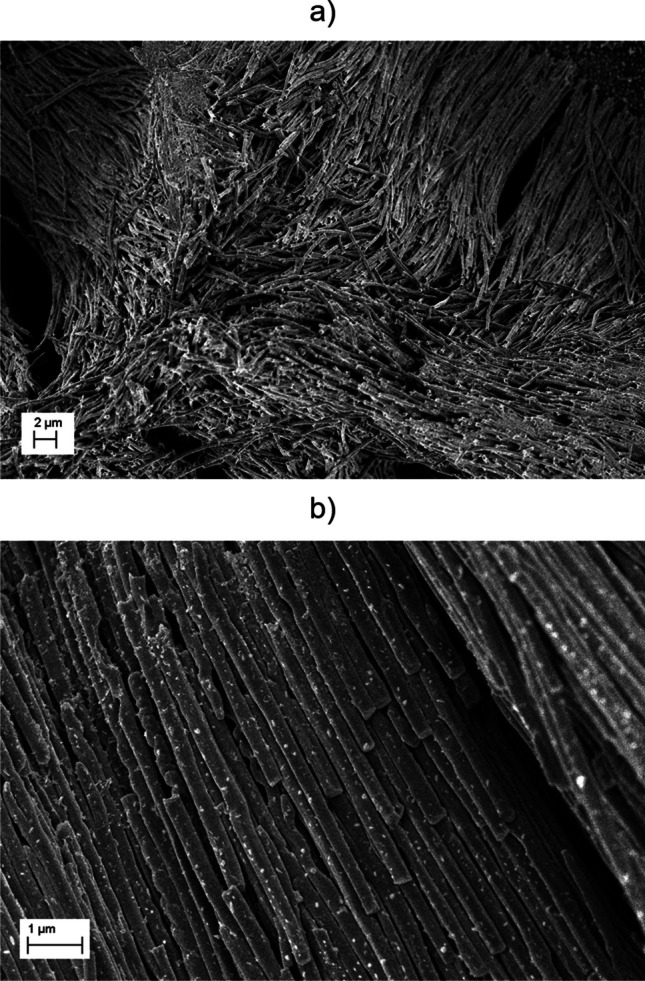
SEM images of the PIN-Au NPs composite, where **a** shows the survey image of the whole surface and **b** shows a detailed image of the structured coating of the GC electrode

Although Raman spectroscopy can be efficiently used in monitoring dyes, its main limitation is the detection limit. From that point of view, the electrochemical approach seems favourable. However, the pigments and organic compound traces are detectable within the proposed protocol. Therefore, the leakage of the chemicals, particularly dyes, from the weathered microplastics was investigated with the cyclic voltammetry CV. The first analyses were performed using the bare GC electrode, while the obtained CV curves did not reveal any peaks that could confirm the leaching of chemicals from the microplastic samples. Therefore, the surface of GC was modified with the electroactive components forming a material with a highly-developed surface made of polyindole nanobrush decorated with Au nanoparticles (PIN-Au NPs). Before electrochemical measurements, the electrode morphology was studied with the SEM technique. As seen in Fig. 5, the electrode is modified with wire-like PIN structures (Fig. 5a), and the PIN is decorated with An NPs to improve the detection limit of compounds resealed from the microplastic-based samples.

Then, the electrochemical measurements were performed at the same conditions as the samples measured using bare GC as the working electrode. Besides all samples investigated with the Raman spectroscopy, only 2 samples revealed the leaching, namely samples 3 and 13. As can be seen, the recorded CV curves, both anodic and cathode peaks, can be distinguished. The CV curves were recorded with different polarization rates from 5 to 40 mV^.^s^−1^ for samples 3 and 5 to 60 mV^.^s^−1^ for sample 13. Interestingly, the curves have a completely different shape. According to sample 3, two anodic peaks are recorded, while the anodic peak at about 0.4–0.5 V comes from overlapping 2 peaks.

**Fig. 6:**
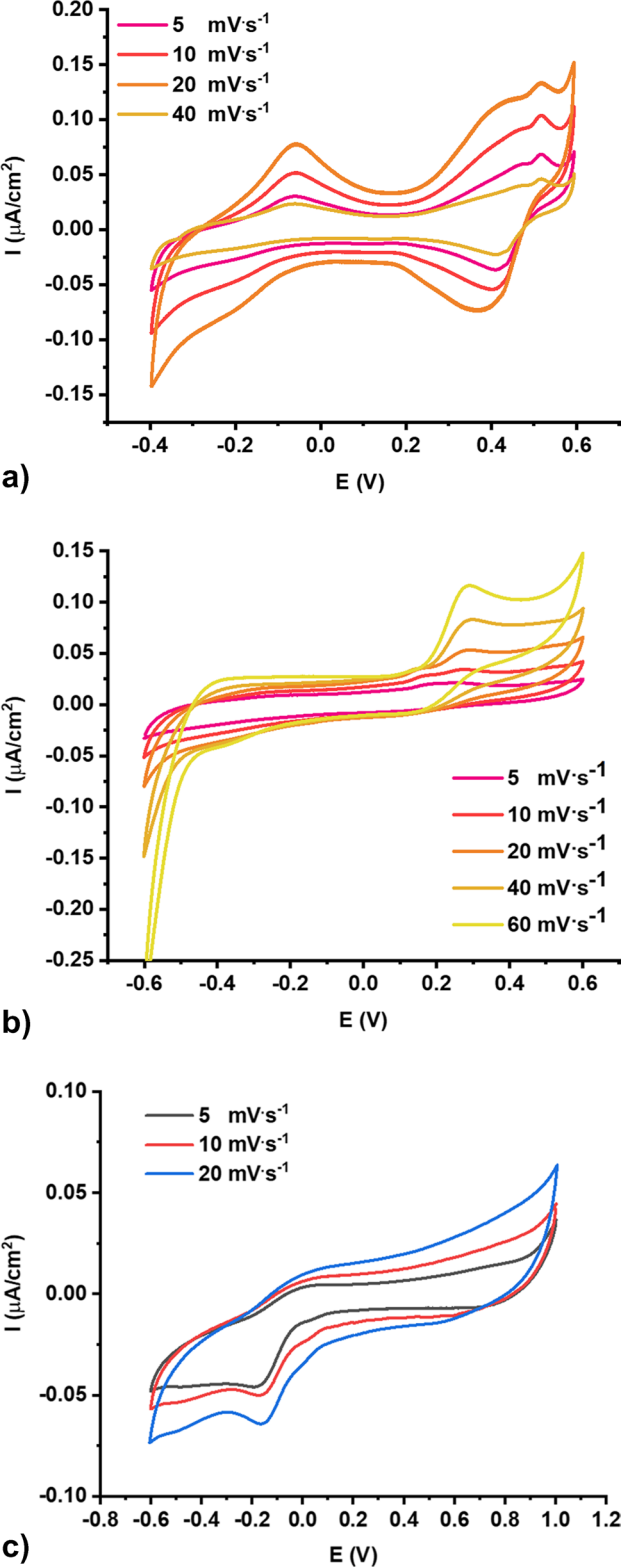
Cyclic voltammetry curves for leaching solution of **a** sample 3 and **b** sample 13 recorded in 0.1 M LiClO_4_ using WE: GC coated with PIN-Au NPs, RE: sat. Ag|AgCl|KCl, CE: Pt mesh, and **c** blanc solution 0.1 M LiClO_4_ exposed on the air

Only one characteristic peak is visible in the 0.3–0.4 V range for the cathodic region, and the curve broadens in the more cathodic potential range. Anodic-cathodic signals below 0.0 V can correspond to some adsorption-desorption, whether it is not clear what compounds are detected. It can come from oxygen, while the solution was purged with nitrogen before the measurements and was also purged above the solution for the whole measurement. Complementarily, the blanc sample with basic electrolyte without leachates was measured, and non-oxygen-based peaks were observed, where non-pair of peaks were recorded. So far, the blanc solution was measured again after its exposition on the air. Figure 6b shows the CV curves, with the characteristic peak corresponding to the oxygen reduction. Its position is in a different potential range than the peak on CV curves for sample 3, so the peaks do not come from the oxygen. Sample 13 also reveals the peak, while the signal has an entirely different shape. The anodic peak at about 0.2–0.3 V is seen, and up to −0.4 V, the curve broadens until the drop after −0.4 V. The drop on the curve could be ascribed to the hydrogen evolution. Both solutions for samples 3 and 13 seem to deliver exciting leaching data. At the same time, the full description of the compounds detected in our studies would need additional techniques, e.g. chromatography towards separating particular compounds and mass spectrometry for their characterization.

Mechanically damaged plastics, especially microplastics (MPs), release many harmful substances into the environment, negatively impacting health and affecting living organisms, leading to numerous disorders — mainly of the endocrine, immune and nervous systems. Many chemicals like plasticizers, flame retardants, sealants and catalysts quickly release MPs into the water and accumulate in a food chain. Besides polycyclic aromatic hydrocarbons, plasticizers, phthalates, bis-phenols, UV-stabilizers, antioxidants and organotins, microplastics release other compounds, including pigments. Although many techniques are efficiently used to detect microplastics, monitoring chemicals released from MPs seems much more complicated due to too little knowledge about the various compounds released into the environment. One of the promising and innovative approaches is electrochemical sensing. Some research results were presented on plastic additives’ pollution in water environments. Each used different methods for measuring each substance’s contamination and identification. This work aims to answer whether cyclic voltammetry efficiently identifies such pollution. For example, this method investigated its usage in identifying bisphenol A (another example of a plastic additive). The research has shown that using a three-dimensional porous silicon-carbon-gold electrode can detect as low substance concentrations as 5.0 × 10–8 M, which offers significant sensitivity to such a system. Another research example has shown that modifying working electrodes (Cu_2_O@C) can determine bisphenol A oxidation processes that cannot be observed using unmodified glassy carbon electrodes. The Cu_2_O@C notation means the electrode was modified with Cu_2_O nanoparticles decorated with amorphous carbon as the core-shell electroactive layer. They achieved the limit of detection (LOD) in 260 nM–31 μM, which shows that this method can detect tiny amounts of that substance.

Furthermore, they mentioned a few other modifications to detect bisphenol A that showed that LOD could reach a single nM. Considering that such chemicals can be freed from plastics in even fewer amounts, this method can be the first to detect such quantities of these substances. It is also worth mentioning that LOD can strongly depend on the type of electrode modification. Electrochemistry offers high effectiveness, low costs, reliability and versatility compared to the classical water pollution monitoring and treatment methods. Moreover, electrochemical methods are fast, portable, user-friendly and can be adapted to different on-site monitoring sites. Literature described its broad application of electrochemical tools for the treatment of heavy metal ions occurring toxicity for living organisms like toxicities of Hg(II), Cd(II), Cr(III), As(III), Pb(II), UO_2_(II), Tl(I), Cr(VI), Ag(I), and Cu(II), removal of surfactants with electrocoagulation, pesticide treatment, pharmaceutical residues electrochemical degradation and removal. Another group of compounds affecting various parts of the environment, including terrestrial and aquatic communities, are dyes released from the textile, food, and synthetic materials industry. Many of these dyes exhibit ecotoxic or genotoxic properties, leading to the deep need for selective and sensitive monitoring. So far, due to the tremendous pollution of water reservoirs with these and other organic compounds, electrochemical methods have been applied. The main techniques used in the electrochemical detection of chemicals include potentiometry, amperometry, conductometry, impedance and voltammetry, including linear sweep voltammetry (LSV), cyclic voltammetry (CV), differential pulse voltammetry (DPV) and anodic stripping voltammetry (ASV). Also, photo-assisted techniques can enhance the detection/removal of chemicals from the solution. Nevertheless, when it comes to the particular chemicals monitoring in the water, the voltammetry-based method is one of the most commonly used (Warczak et al. 2021). However, depending on the chemistry of the electrode, experimental conditions and the chosen method, the detection limit of dye and its degradation (and photodegradation) products can be increased.

Among many organic dyes that can be released from plastics, they negatively impact health. One such example is Congo red, which adversely affects health. Shetti et al. indicate the risk of developing cancer or even anaphylactic shock after exposure to such dye (Shetti et al. 2019). They refer to the electrochemical detection of the Congo red ad its degradation within the electrode modified with graphene oxide while also presenting its detection by use of the classical glassy carbon electrode, where the differential pulse voltammetry (DPV), linear sweep voltammetry (LSV) and cyclin voltammetry (CV) were used. Ghalkhani et al. present (Ghalkhani et al. 2022) electrochemical sensing of food colourant Ponceau dyes occurring cytotoxicity along with their removal and degradation. Another piece of literature describes the removal and detection within the electrochemical oxidation of the indigo textile dye. The following work presented by Tran et al. has shown the voltammetric detection of Sunset yellow dye using a carbon-based electrode (Tran et al. 2019). Ghoreishi et al. (2013) investigated the sensing of Red 10B and tartrazine dyes within the cyclic voltammetry by using a nanostructural gold-modified carbon nanotubed-based electrode, while Ghoreishi et al. (2011) also presented voltammetry to be used for tartrazine dye detection.

Other studies refer to the application of electrochemical tools, including CV for the detection of alizarin red (AR), erythrosine, Cibacron Blue F3GA, crystal violet, Turquoise blue 15 (AT15), Sudan I and Sudan II, Procion Red HE-3B 9 (RR120) and Procion Green HE-4BD (RG19), Rhodamine B, Olive Green B dye (VG3), Reactive Black 5 (RB5), Red 231 (Radi et al. 2013), carminosine and Disperse Red 13 (DR13), where all these dyes have a negative impact on health. To our knowledge, this study reports as a first in the feasibility of an electrochemical approach to tackle the issue of compound leakage from aged microplastics. Interestingly, electrochemical methods are also used to analyse biofilm related to the PE (Wang et al. 2022).

## Conclusions and future perspectives

The microplastics onshore in three ports on the Central and Western Meditteranean Sea (Saint-Tropez, Portoferraio and Porto Ercole) and four selected locations (two within the Porto Ercole) were studied. Polyethylene was the dominant fraction in all places except the beach at Porto Ercole. The Raman spectroscopy proved to be an efficient tool in the direct qualitative characterization of MPs, enabling the 94% identification level without additional pretreatment. From the various colours, one can conclude about substantially different origins of MPs. Moreover, their rough and cracked surface indicates their natural ageing.

Thus, the leakage problem may occur spontaneously in environmental conditions. Considering that added compounds are primarily responsible for the toxic effect, we stressed this aspect within the presented study. In some samples weathered in the laboratory, the leakage of compounds was observed within the electrochemical tools. Cyclic voltammetry curves show a complex composition of the solution. They need a deeper investigation of the leaking compounds in different conditions, including pH, temperature, oxygen saturation, ionic strength and presence of UV light while weathering towards the simulation of different environmental conditions. The analyses can also be combined with different techniques to identify the released compounds precisely. The obtained results show that just a week of weathering is sufficient to release some microplastic chemicals. An innovative electrochemical approach to the problem of leaking compounds can enhance the detection limit, which was one of the main obstacles in efficiently monitoring this kind of pollution. Our research has the following limitations: simplified sampling and preliminary electrochemical identification. Those current problems will be addressed in the near future.

### Supplementary information

ESM 1 (DOCX 19.0 MB)

## Data Availability

The datasets obtained during the current study are available from the corresponding author on a reasonable request.
