# Supplementary material for: The comparative study by Raman spectroscopy of the plastic tide in the three ports of the Mediterranean Sea
Source: Environ Sci Pollut Res Int. 2023 Nov 24;30(59):124093–105. doi: 10.1007/s11356-023-30973-z (PMC10746617; doi:10.1007/s11356-023-30973-z)
Supplement: Supplementary file 1 — (DOCX 19.0 MB) [file 11356_2023_30973_MOESM1_ESM.docx]

**Supplementary Data**

**Table S1** The macroscopic characteristics of debris from Portofferraio selected for the electrochemical measurements.

| Number of sample | Characteristics |
| --- | --- |
| **37** | light blue with discolored areas, quite regular shape, straight edges |
| **52** | yellow, irregular shape, rounded egdes |
| **21** | dark blue, thin with rounded external edge |
| **36** | very light blue, regular shape, straight edges, really small probe |
| **35** | white, regular, triangular shape |
| **50** | red, irregular shape, curved at about half of its length |
| **3** | dark red with discolored areas, triangular shape with some irregularities |
| **48** | light purple tube, jagged at one of its ends |
| **49** | yellow, irregular shape with sharp edge at one side |
| **7** | dark green with discolored areas, irregular shape, with sharp edge at one side |
| **47** | white, rounded |
| **18** | dark green, regular shape, really small probe |
| **30** | blue, irregular shape, blunt egde at one side and symetrically cut on the other (sharp edge) |
| **22** | light blue, light passes through probe, irregular shape with two rounded edges |
| **13** | green, regular shape with small appendix at one vertex |

**Table S2** Summary of collected probes divided into their origin locations and their percentage participation in overall.

|  | **Portoferraio** | **Porto Ercole loc. 1** | **Porto Ercole loc. 2** | **Saint Tropez** | **Sum** | **Overall percentege**  **[%]** |
| --- | --- | --- | --- | --- | --- | --- |
| **PE** | 34 | - | 21 | 17 | 72 | 47 |
| **PP** | 20 | 1 | 11 | 4 | 36 | 24 |
| **PS** | - | 13 | 1 | - | 14 | 9 |
| **PMMA** | - | 2 | 1 | 1 | 3 | 2 |
| **PA** | - | 2 | - | - | 2 | 1 |
| **not identified** | 1 | 6 | 1 | 1 | 9 | 6 |
| **other** | - | 5 | 8 | 4 | 17 | 11 |


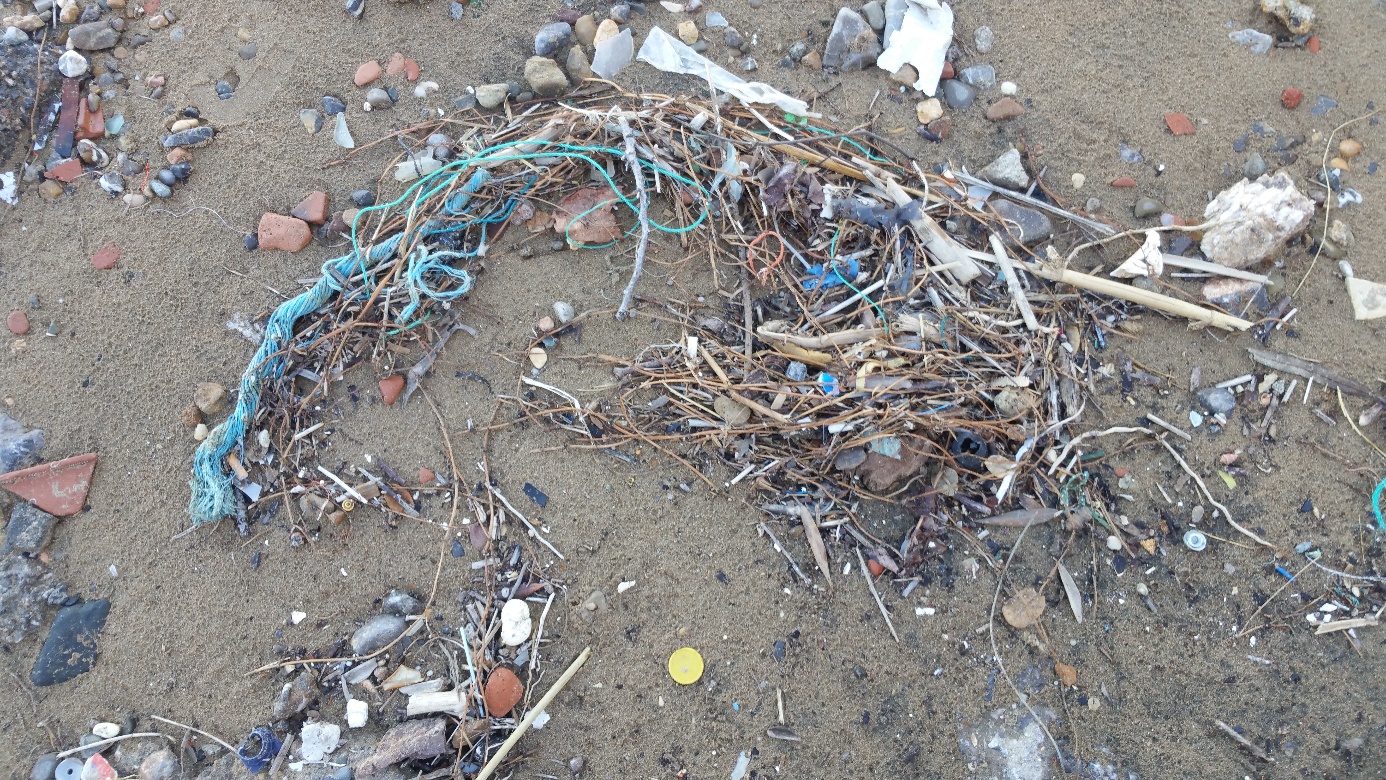


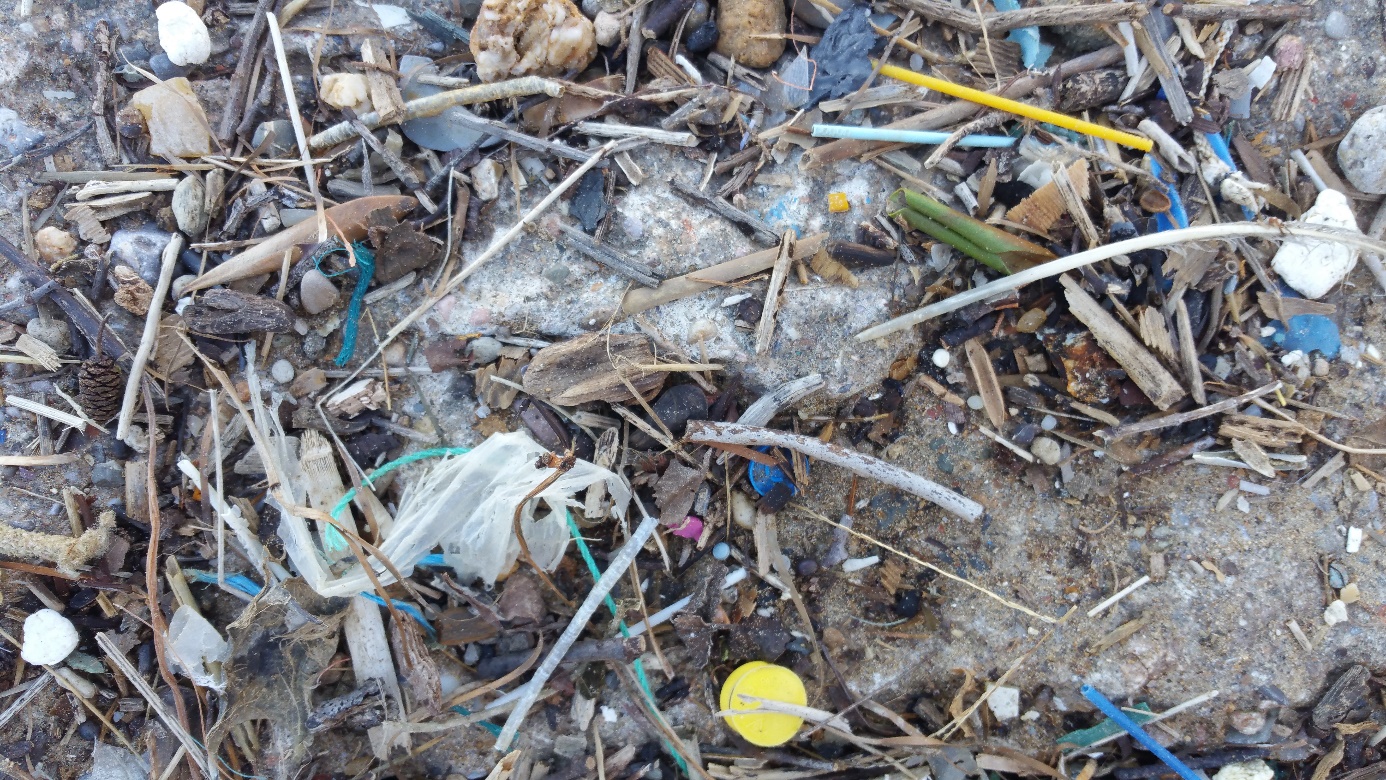


**Figure S1.** The macroscopic overview of beaches in Porto Ercole


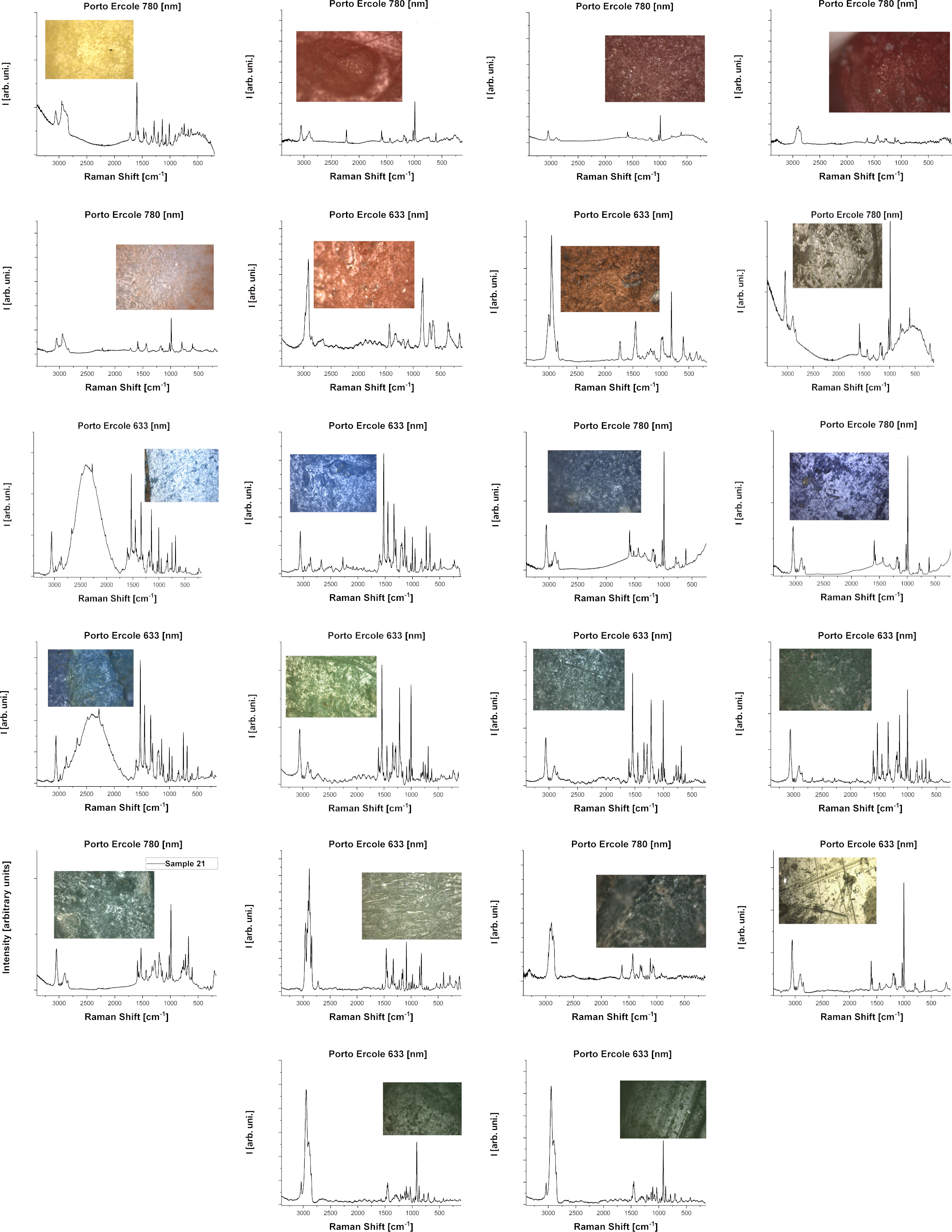
Figure S2. Raman spectra and images for probes collected at localisation 1 in Porto Ercole


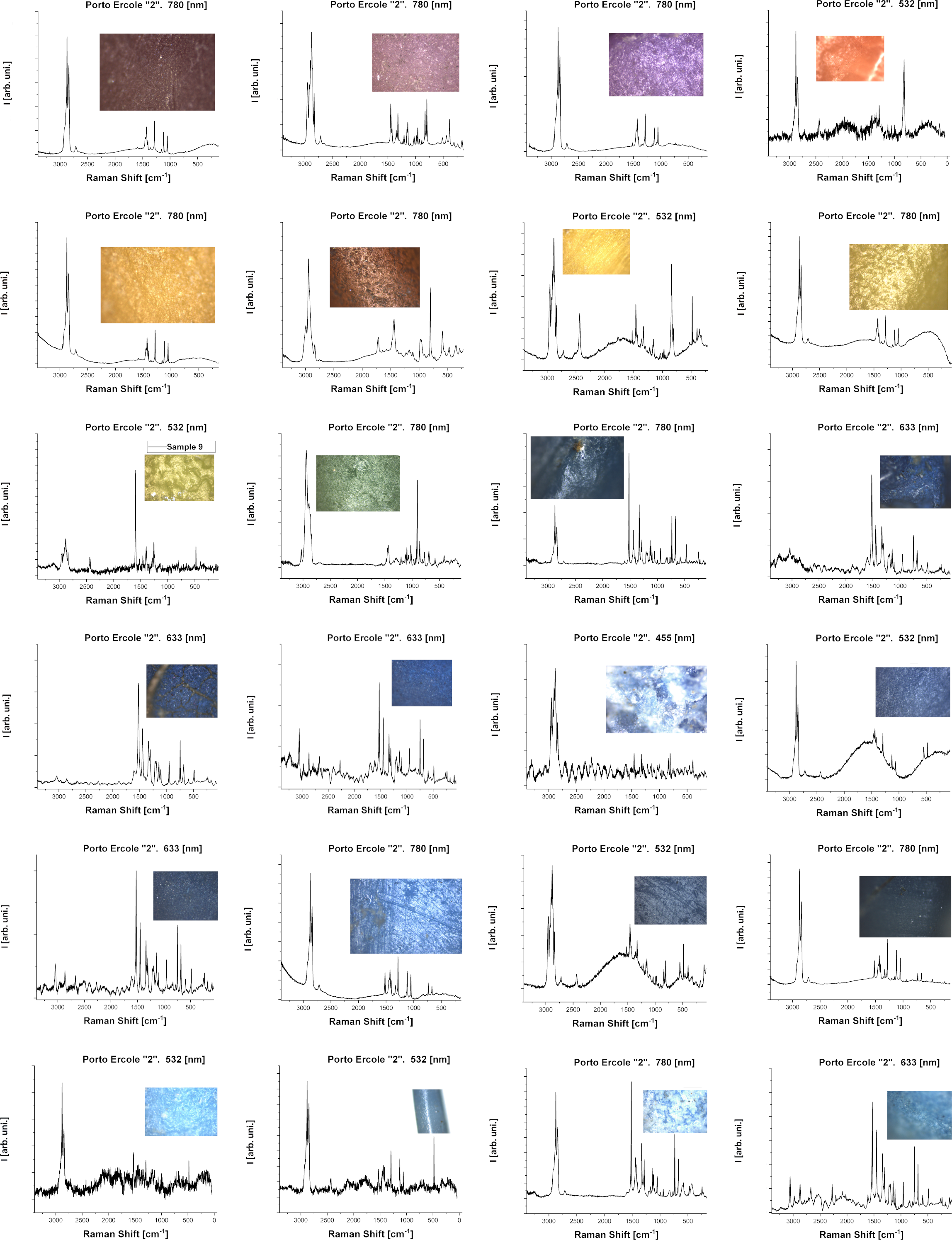


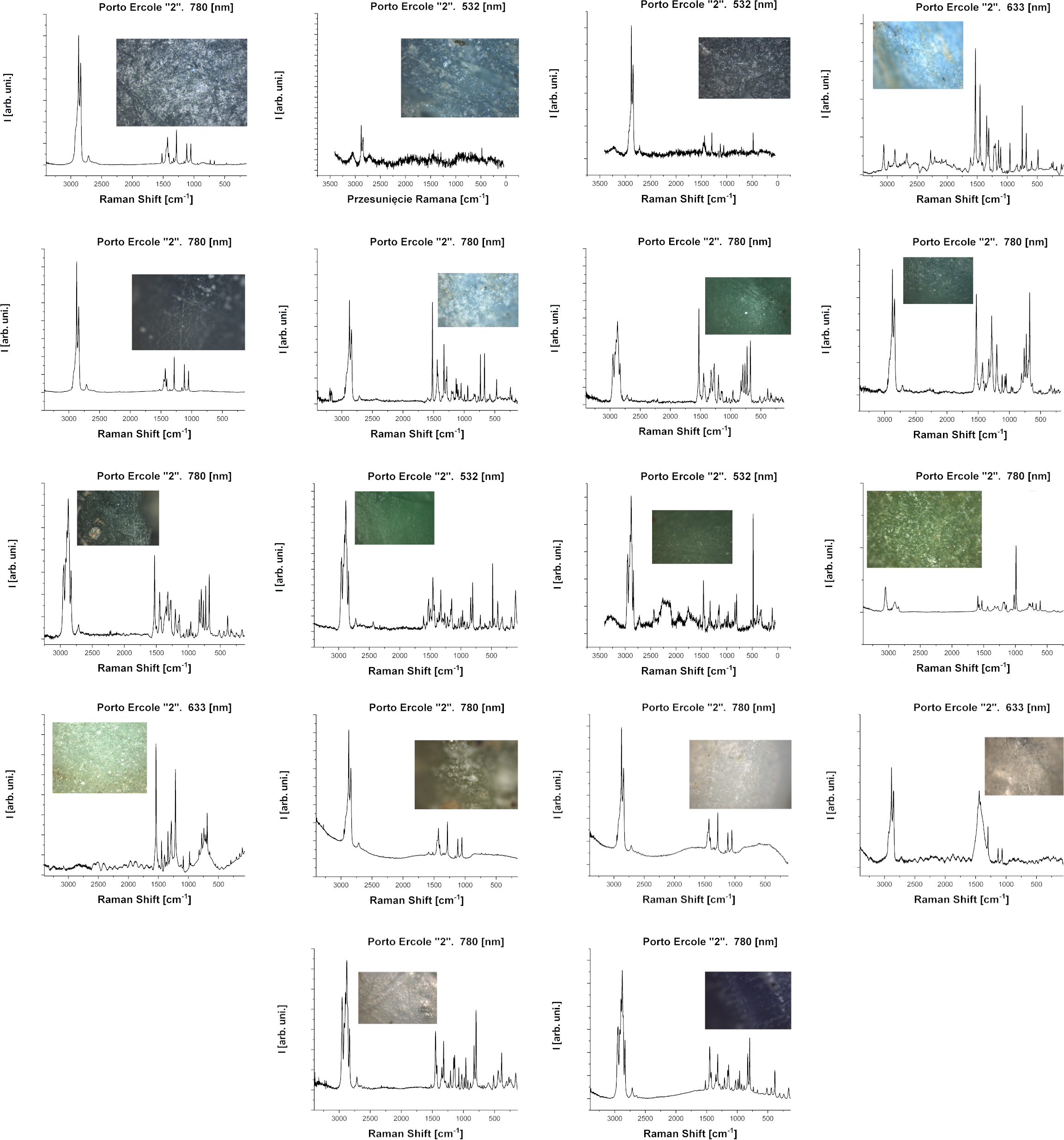
Figure S3. Raman spectra of probes collected at location 2 in Porto Ercole


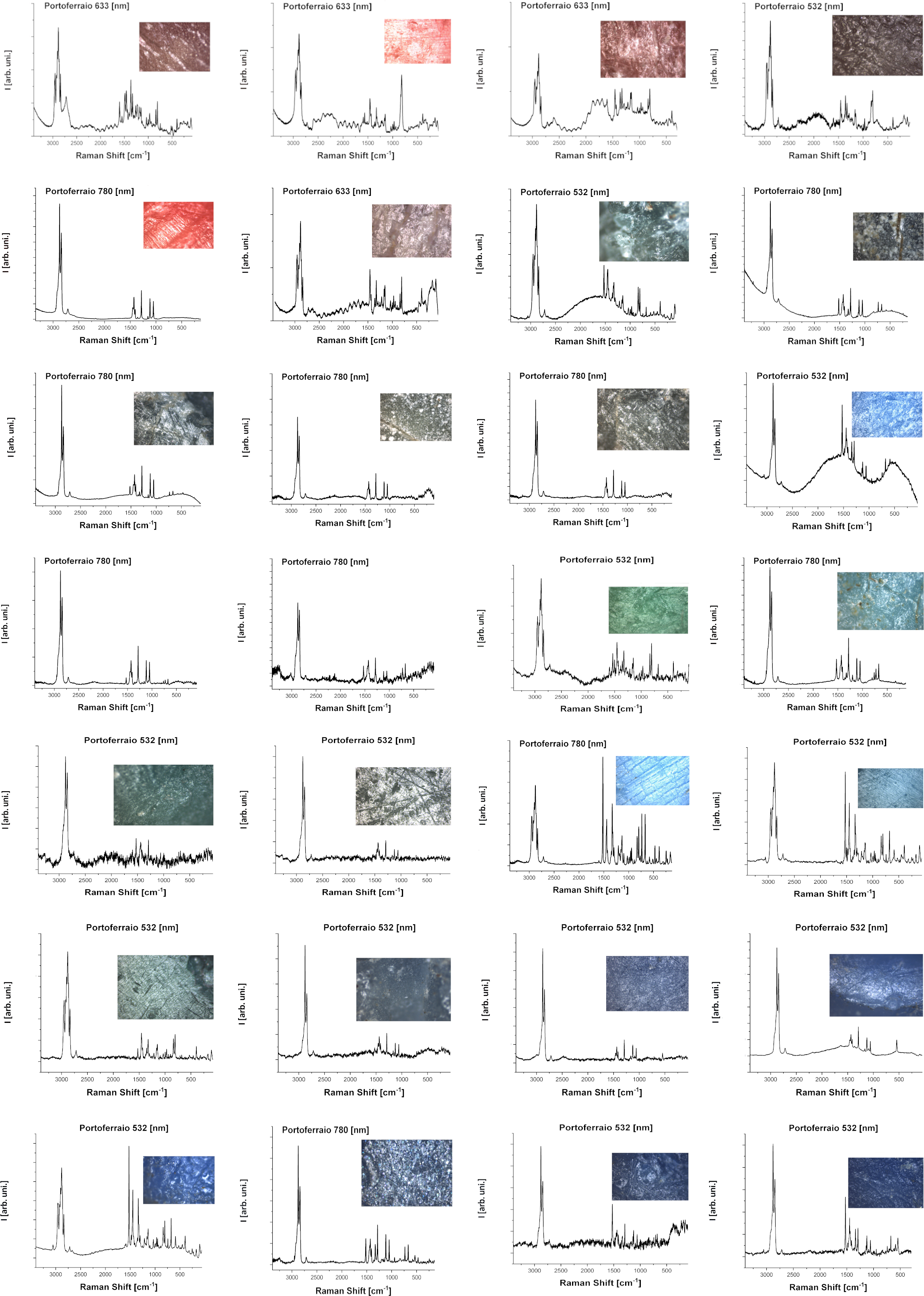


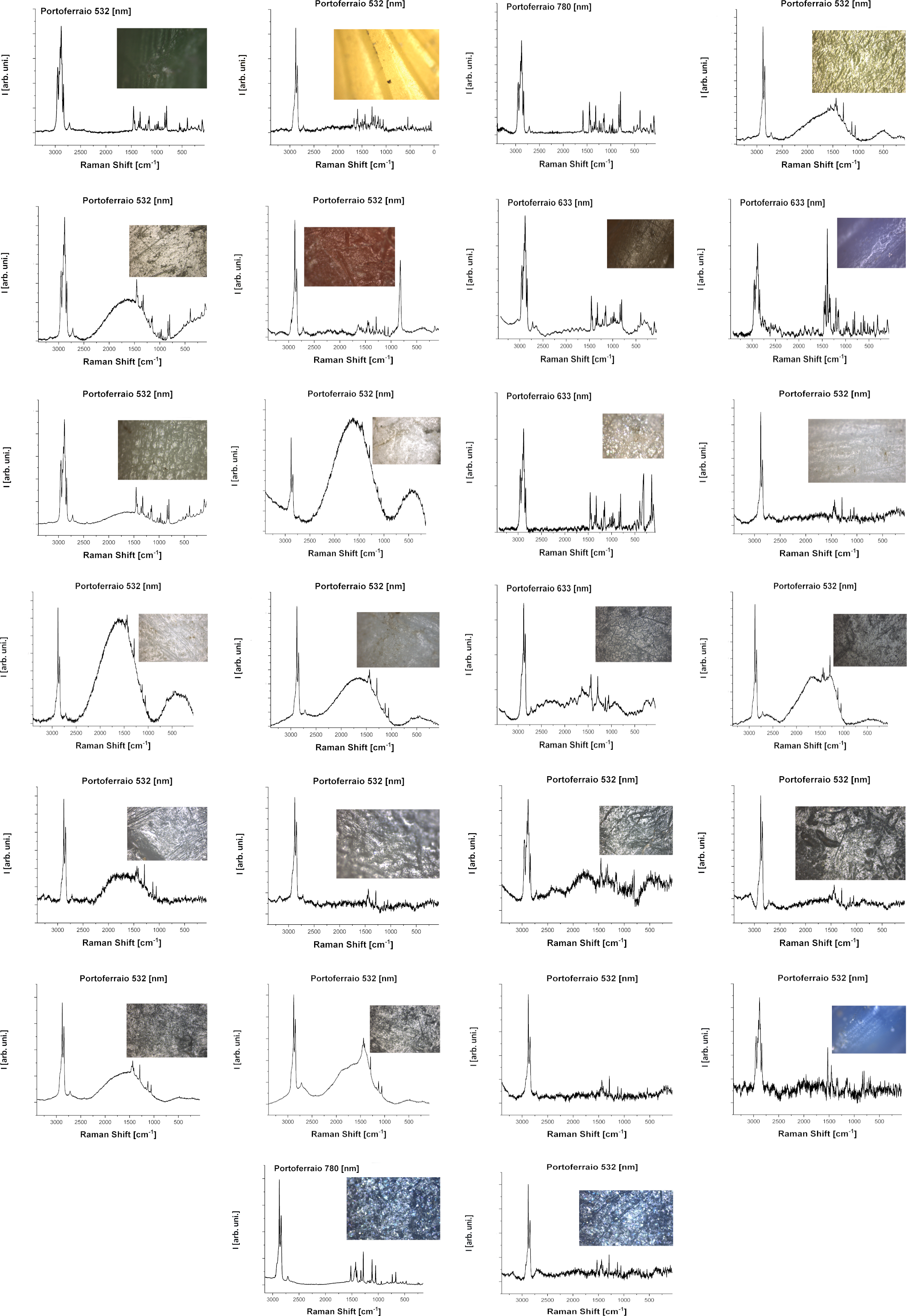
Figure S4. Raman spectra and images of probes collected in Portoferraio.


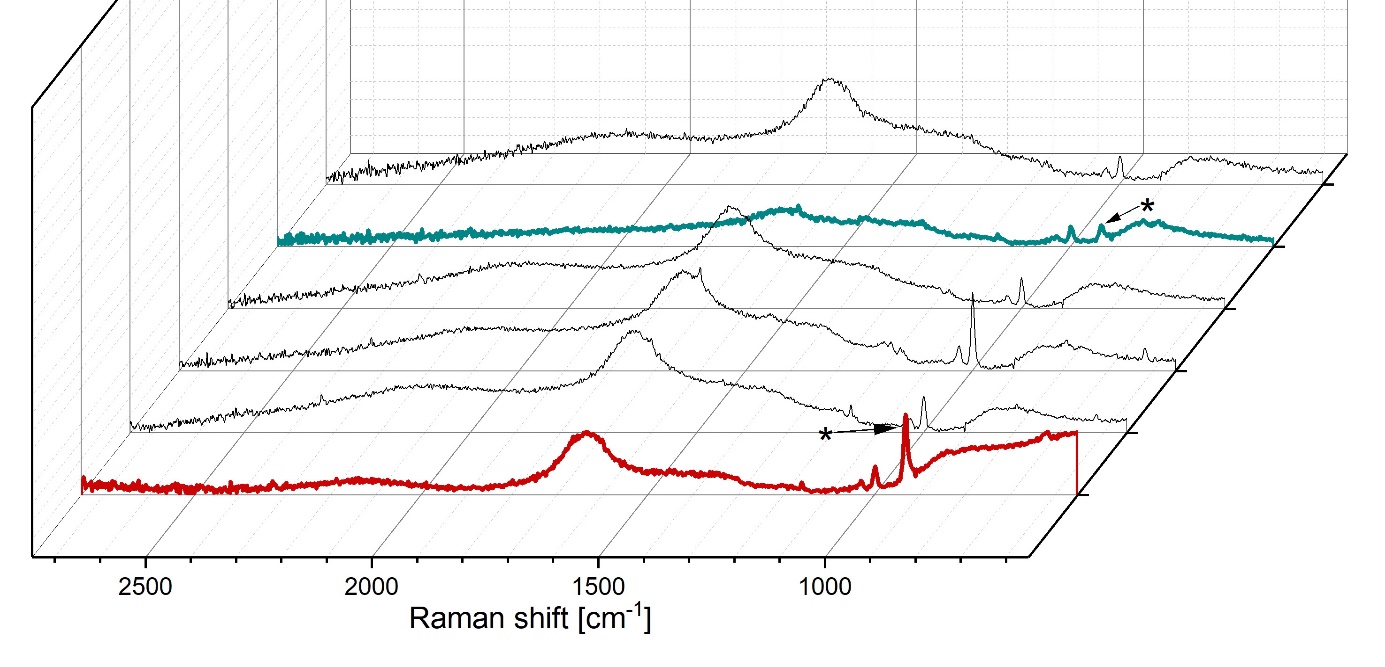


Figure S5. Two bands present in water as clear signs of leakage in samples 3 (PP, red) and 13 (PE, green) from Portoferraio.


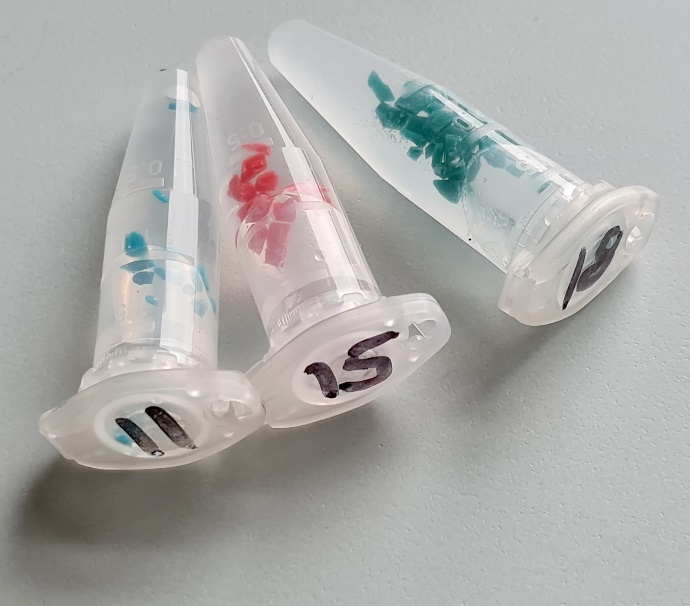


Figure S6**.** An example of samples from Portoferraio aged in a milli-Q water for over 10 months
